# *cis*-Dichloridobis(1,10-phenanthroline)chromium(III) chloride

**DOI:** 10.1107/S1600536810053845

**Published:** 2011-01-08

**Authors:** Xiaoli Gao

**Affiliations:** a Taiyuan Normal Colleage, Department of Chemistry, Taiyuan, Shanxi 030006, People’s Republic of China

## Abstract

In the title complex, [CrCl_2_(C_12_H_8_N_2_)_2_]Cl, the Cr^III^ ion is situated on a twofold rotation axis and displays a slightly distorted octa­hedral CrCl_2_N_4_ coordination geometry. The Cr environment is composed of a *cis* arrangement of two 1,10-phenanthroline and two chloride ligands. The chloride counter-anion exhibits half-occupation and is equally disordered over two positions.

## Related literature

For background to chromium(III) complexes, see: Vincent (2000 ▶). For the structure of a related Cr(III) complex with phenanthroline ligands, see: Birk *et al.* (2008 ▶).
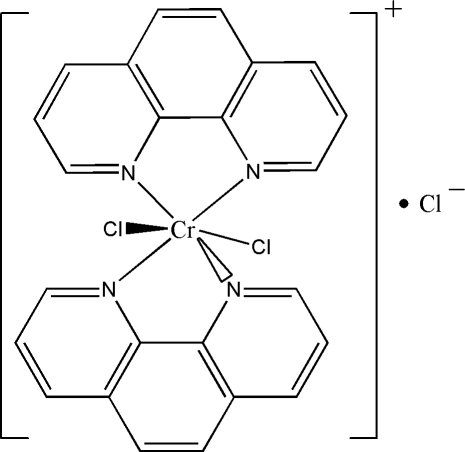


## Experimental

### 

#### Crystal data

[CrCl_2_(C_12_H_8_N_2_)_2_]Cl
*M* *_r_* = 518.76
Monoclinic, 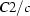

*a* = 15.446 (7) Å
*b* = 13.762 (6) Å
*c* = 12.536 (5) Å
β = 100.398 (6)°
*V* = 2621.1 (19) Å^3^
*Z* = 4
Mo *K*α radiation
μ = 0.76 mm^−1^
*T* = 298 K
0.40 × 0.10 × 0.10 mm

#### Data collection

Bruker SMART CCD area-detector diffractometer
Absorption correction: multi-scan (*SADABS*; Sheldrick, 2000 ▶) *T*_min_ = 0.751, *T*_max_ = 0.928
5182 measured reflections
2265 independent reflections
1778 reflections with *I* > 2σ(*I*)
*R*_int_ = 0.039

#### Refinement

*R*[*F* ^2^ > 2σ(*F* ^2^)] = 0.070
*wR*(*F* ^2^) = 0.211
*S* = 1.07
2265 reflections
150 parameters
H-atom parameters constrained
Δρ_max_ = 0.92 e Å^−3^
Δρ_min_ = −0.35 e Å^−3^

### 

Data collection: *SMART* (Bruker, 2000 ▶); cell refinement: *SAINT* (Bruker, 2000 ▶); data reduction: *SAINT*; program(s) used to solve structure: *SHELXS97* (Sheldrick, 2008 ▶); program(s) used to refine structure: *SHELXL97* (Sheldrick, 2008 ▶); molecular graphics: *SHELXTL* (Sheldrick, 2008 ▶); software used to prepare material for publication: *SHELXTL*.

## Supplementary Material

Crystal structure: contains datablocks I, global. DOI: 10.1107/S1600536810053845/wm2436sup1.cif

Structure factors: contains datablocks I. DOI: 10.1107/S1600536810053845/wm2436Isup2.hkl

Additional supplementary materials:  crystallographic information; 3D view; checkCIF report

## Figures and Tables

**Table 1:** Selected bond lengths (Å)

Cr1—N1	2.062 (4)
Cr1—N2	2.073 (4)
Cr1—Cl1	2.2941 (15)

**Fig. 1.**
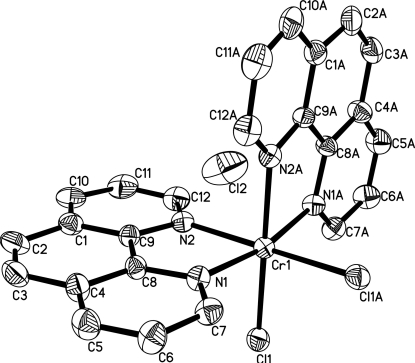
The molecular structure of the title compound, showing the atom-numbering scheme. Displacement ellipsoids are drawn at the 30% probability level; H atoms were omitted for clarity. [Symmetry code A: -x + 1, y, -z + 3/2.]

**Fig. 2.**
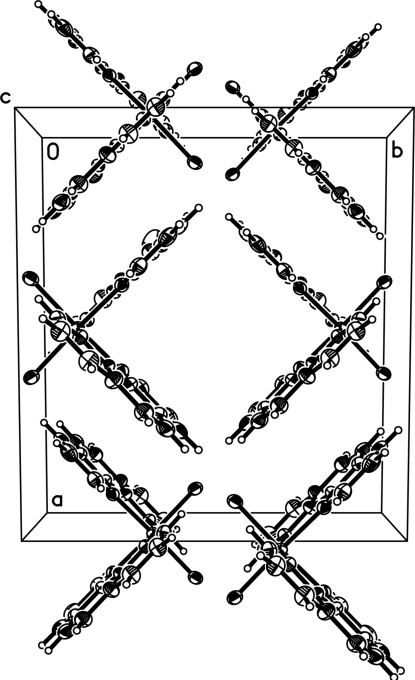
The crystal structure of title compound, projected along the c-axis.

## supplementary crystallographic information

### Comment

Chromium is known to activate enzymes, maintain protein stability and enhance carbohydrate metabolism. Organic chromium (III) sources have been shown to enhance the availability of chromium. Nutritionists believe organic chromium should be supplemented in most animal diets. A search has therefore been underway to identify the biologically active form of chromium, *viz.* the biomolecule that binds chromium(III) and possesses an intrinsic function associated with insulin action in mammals (Vincent, 2000). We designed and synthesized a new chromium complex, [CrCl_2_(C_12_H_8_N_2_)_2_]Cl.

The Cr atom has site symmetry 2 and exhibits a distorted octahedral coordination, defined of four N atoms [Cr—N1: 2.062 (4) Å, Cr—N2: 2.073 (4) Å] from two symmetry-related 1,10-phenanthroline ligands and of two symmetry-related Cl ligands [2.2941 (15) Å]. The ligands are in a *cis* arrangement (Fig. 1.). The two planar 1,10-phenanthroline ligands show an approximate perpendicular orientation to each other [N1—Cr1—N2A: 91.81 (14) °, N2—Cr1—N2A: 88.5 (2) °]. These values are similar to that of the structure of the related compound *cis*-difluoridobis(1,10-phenanthroline)chromium(III) perchlorate monohydrate (Birk *et al.*, 2008).

The crystal packing of the title compound is displayed in Fig. 2.

### Experimental

The title compound was prepared by the following method. CrCl_3_^.^6H_2_O (0.665 g, 0.0025 mol) and zinc powder (0.1 g, 0.0015 mol) were added into methanol (30 ml) and then refluxed for 1 h. To the mixture 1,10-phenanthroline (0.99 g, 0.0050 mol) in methanol (20 ml) was added dropwise and allowed to reflux for another 30 min. The solution was put aside and X-ray quality crystals were obtained after two days at room temperature.

### Refinement

The H atoms were treated as riding atoms, with C—H (CH) = 0.93 Å, and with *U*_iso_(H) = 1.5 *U*_eq_ (parent atom).

### Crystal data

**Table:**

[CrCl_2_(C_12_H_8_N_2_)_2_]Cl	*F*(000) = 1052
*M**_r_* = 518.76	*D*_x_ = 1.315 Mg m^−^^3^
Monoclinic, *C*2/*c*	Mo *K*α radiation, λ = 0.71073 Å
Hall symbol: -C 2yc	Cell parameters from 1354 reflections
*a* = 15.446 (7) Å	θ = 2.7–19.9°
*b* = 13.762 (6) Å	µ = 0.76 mm^−^^1^
*c* = 12.536 (5) Å	*T* = 298 K
β = 100.398 (6)°	Block, red
*V* = 2621.1 (19) Å^3^	0.40 × 0.10 × 0.10 mm
*Z* = 4	

### Data collection

**Table:**

Bruker SMART CCD area-detector diffractometer	2265 independent reflections
Radiation source: fine-focus sealed tube	1778 reflections with *I* > 2σ(*I*)
graphite	*R*_int_ = 0.039
φ and ω scans	θ_max_ = 25.0°, θ_min_ = 2.4°
Absorption correction: multi-scan (*SADABS*; Sheldrick, 2000)	*h* = −18→18
*T*_min_ = 0.751, *T*_max_ = 0.928	*k* = −16→16
5182 measured reflections	*l* = −7→14

### Refinement

**Table:**

Refinement on *F*^2^	Primary atom site location: structure-invariant direct methods
Least-squares matrix: full	Secondary atom site location: difference Fourier map
*R*[*F*^2^ > 2σ(*F*^2^)] = 0.070	Hydrogen site location: inferred from neighbouring sites
*wR*(*F*^2^) = 0.211	H-atom parameters constrained
*S* = 1.07	*w* = 1/[σ^2^(*F*_o_^2^) + (0.1164*P*)^2^ + 5.5982*P*] where *P* = (*F*_o_^2^ + 2*F*_c_^2^)/3
2265 reflections	(Δ/σ)_max_ < 0.001
150 parameters	Δρ_max_ = 0.92 e Å^−^^3^
0 restraints	Δρ_min_ = −0.35 e Å^−^^3^

### Special details

**Table:**

Experimental. the Cl-counter anion is only half occupied and equally disordered over two positions.
Geometry. All e.s.d.'s (except the e.s.d. in the dihedral angle between two l.s. planes) are estimated using the full covariance matrix. The cell e.s.d.'s are taken into account individually in the estimation of e.s.d.'s in distances, angles and torsion angles; correlations between e.s.d.'s in cell parameters are only used when they are defined by crystal symmetry. An approximate (isotropic) treatment of cell e.s.d.'s is used for estimating e.s.d.'s involving l.s. planes.
Refinement. Refinement of *F*^2^ against ALL reflections. The weighted *R*-factor *wR* and goodness of fit *S* are based on *F*^2^, conventional *R*-factors *R* are based on *F*, with *F* set to zero for negative *F*^2^. The threshold expression of *F*^2^ > σ(*F*^2^) is used only for calculating *R*-factors(gt) *etc*. and is not relevant to the choice of reflections for refinement. *R*-factors based on *F*^2^ are statistically about twice as large as those based on *F*, and *R*- factors based on ALL data will be even larger.

### Fractional atomic coordinates and isotropic or equivalent isotropic displacement parameters (Å^2^)

**Table:**

	*x*	*y*	*z*	*U*_iso_*/*U*_eq_	Occ. (<1)
Cr1	0.5000	0.83468 (7)	0.7500	0.0338 (4)	
Cl1	0.60948 (7)	0.94883 (9)	0.76093 (10)	0.0481 (4)	
N1	0.5196 (2)	0.8199 (3)	0.9163 (3)	0.0386 (9)	
N2	0.5952 (2)	0.7267 (3)	0.7714 (3)	0.0383 (9)	
C1	0.6878 (3)	0.6322 (4)	0.9120 (4)	0.0467 (12)	
C2	0.7141 (4)	0.6151 (4)	1.0254 (5)	0.0600 (14)	
H2	0.7577	0.5695	1.0490	0.072*	
C3	0.6771 (4)	0.6636 (4)	1.0997 (5)	0.0576 (14)	
H3	0.6957	0.6507	1.1731	0.069*	
C4	0.6099 (3)	0.7344 (4)	1.0672 (4)	0.0495 (12)	
C5	0.5685 (4)	0.7889 (4)	1.1387 (4)	0.0582 (14)	
H5	0.5837	0.7789	1.2131	0.070*	
C6	0.5068 (4)	0.8555 (4)	1.0999 (4)	0.0555 (14)	
H6	0.4806	0.8923	1.1475	0.067*	
C7	0.4820 (3)	0.8693 (4)	0.9869 (4)	0.0478 (12)	
H7	0.4381	0.9142	0.9612	0.057*	
C8	0.5826 (3)	0.7539 (3)	0.9552 (4)	0.0380 (10)	
C9	0.6226 (3)	0.7021 (3)	0.8779 (4)	0.0370 (10)	
C10	0.7257 (4)	0.5865 (4)	0.8319 (5)	0.0588 (14)	
H10	0.7682	0.5387	0.8506	0.071*	
C11	0.6995 (4)	0.6130 (5)	0.7255 (5)	0.0650 (16)	
H11	0.7250	0.5839	0.6718	0.078*	
C12	0.6345 (4)	0.6836 (4)	0.6980 (5)	0.0531 (13)	
H12	0.6180	0.7012	0.6255	0.064*	
Cl2	0.6999 (6)	0.6655 (4)	0.4261 (5)	0.157 (3)	0.50

### Atomic displacement parameters (Å^2^)

**Table:**

	*U*^11^	*U*^22^	*U*^33^	*U*^12^	*U*^13^	*U*^23^
Cr1	0.0324 (6)	0.0364 (6)	0.0334 (6)	0.000	0.0081 (4)	0.000
Cl1	0.0390 (7)	0.0488 (7)	0.0549 (8)	−0.0079 (5)	0.0043 (5)	0.0066 (5)
N1	0.040 (2)	0.041 (2)	0.037 (2)	−0.0041 (17)	0.0121 (17)	−0.0010 (16)
N2	0.0376 (19)	0.039 (2)	0.040 (2)	0.0022 (16)	0.0107 (16)	0.0015 (17)
C1	0.040 (3)	0.047 (3)	0.053 (3)	0.002 (2)	0.007 (2)	0.006 (2)
C2	0.053 (3)	0.062 (3)	0.061 (3)	0.012 (3)	−0.001 (3)	0.015 (3)
C3	0.057 (3)	0.064 (4)	0.046 (3)	0.000 (3)	−0.005 (3)	0.010 (3)
C4	0.051 (3)	0.055 (3)	0.041 (3)	−0.009 (2)	0.004 (2)	−0.001 (2)
C5	0.070 (4)	0.068 (4)	0.037 (3)	−0.005 (3)	0.009 (2)	0.001 (3)
C6	0.069 (3)	0.062 (3)	0.039 (3)	−0.002 (3)	0.020 (3)	−0.012 (2)
C7	0.054 (3)	0.047 (3)	0.045 (3)	0.004 (2)	0.016 (2)	−0.005 (2)
C8	0.035 (2)	0.041 (2)	0.038 (2)	−0.0057 (19)	0.0053 (18)	0.0008 (19)
C9	0.034 (2)	0.036 (2)	0.041 (2)	−0.0036 (18)	0.0070 (19)	0.001 (2)
C10	0.052 (3)	0.055 (3)	0.071 (4)	0.021 (3)	0.016 (3)	0.008 (3)
C11	0.070 (4)	0.064 (4)	0.068 (4)	0.025 (3)	0.031 (3)	−0.004 (3)
C12	0.059 (3)	0.057 (3)	0.047 (3)	0.011 (3)	0.020 (3)	−0.002 (2)
Cl2	0.265 (8)	0.125 (4)	0.101 (4)	−0.037 (5)	0.086 (5)	−0.027 (3)

### Geometric parameters (Å, °)

**Table:**

Cr1—N1	2.062 (4)	C3—C4	1.428 (8)
Cr1—N1^i^	2.062 (4)	C3—H3	0.9300
Cr1—N2^i^	2.073 (4)	C4—C5	1.408 (8)
Cr1—N2	2.073 (4)	C4—C8	1.417 (7)
Cr1—Cl1	2.2941 (15)	C5—C6	1.348 (8)
Cr1—Cl1^i^	2.2941 (15)	C5—H5	0.9300
N1—C7	1.330 (6)	C6—C7	1.411 (7)
N1—C8	1.356 (6)	C6—H6	0.9300
N2—C12	1.330 (6)	C7—H7	0.9300
N2—C9	1.368 (6)	C8—C9	1.431 (6)
C1—C10	1.399 (8)	C10—C11	1.372 (8)
C1—C9	1.403 (7)	C10—H10	0.9300
C1—C2	1.426 (8)	C11—C12	1.395 (8)
C2—C3	1.353 (8)	C11—H11	0.9300
C2—H2	0.9300	C12—H12	0.9300
			
N1—Cr1—N1^i^	168.7 (2)	C4—C3—H3	119.5
N1—Cr1—N2^i^	91.81 (14)	C5—C4—C8	116.2 (5)
N1^i^—Cr1—N2^i^	80.07 (15)	C5—C4—C3	124.9 (5)
N1—Cr1—N2	80.07 (15)	C8—C4—C3	118.9 (5)
N1^i^—Cr1—N2	91.81 (14)	C6—C5—C4	120.4 (5)
N2^i^—Cr1—N2	88.5 (2)	C6—C5—H5	119.8
N1—Cr1—Cl1	92.00 (11)	C4—C5—H5	119.8
N1^i^—Cr1—Cl1	95.73 (11)	C5—C6—C7	120.0 (5)
N2^i^—Cr1—Cl1	175.09 (11)	C5—C6—H6	120.0
N2—Cr1—Cl1	89.14 (11)	C7—C6—H6	120.0
N1—Cr1—Cl1^i^	95.73 (11)	N1—C7—C6	121.8 (5)
N1^i^—Cr1—Cl1^i^	92.00 (11)	N1—C7—H7	119.1
N2^i^—Cr1—Cl1^i^	89.14 (11)	C6—C7—H7	119.1
N2—Cr1—Cl1^i^	175.09 (10)	N1—C8—C4	123.3 (4)
Cl1—Cr1—Cl1^i^	93.56 (8)	N1—C8—C9	117.4 (4)
C7—N1—C8	118.3 (4)	C4—C8—C9	119.3 (4)
C7—N1—Cr1	128.4 (3)	N2—C9—C1	123.1 (4)
C8—N1—Cr1	113.2 (3)	N2—C9—C8	116.2 (4)
C12—N2—C9	117.7 (4)	C1—C9—C8	120.6 (4)
C12—N2—Cr1	129.2 (3)	C11—C10—C1	119.4 (5)
C9—N2—Cr1	113.0 (3)	C11—C10—H10	120.3
C10—C1—C9	117.3 (5)	C1—C10—H10	120.3
C10—C1—C2	124.2 (5)	C10—C11—C12	119.9 (5)
C9—C1—C2	118.5 (5)	C10—C11—H11	120.1
C3—C2—C1	121.7 (5)	C12—C11—H11	120.1
C3—C2—H2	119.1	N2—C12—C11	122.5 (5)
C1—C2—H2	119.1	N2—C12—H12	118.7
C2—C3—C4	121.1 (5)	C11—C12—H12	118.7
C2—C3—H3	119.5		
			
N1^i^—Cr1—N1—C7	−137.2 (4)	Cr1—N1—C7—C6	−175.5 (4)
N2^i^—Cr1—N1—C7	−93.5 (4)	C5—C6—C7—N1	−1.8 (8)
N2—Cr1—N1—C7	178.4 (4)	C7—N1—C8—C4	0.4 (7)
Cl1—Cr1—N1—C7	89.6 (4)	Cr1—N1—C8—C4	177.3 (4)
Cl1^i^—Cr1—N1—C7	−4.2 (4)	C7—N1—C8—C9	−179.5 (4)
N1^i^—Cr1—N1—C8	46.3 (3)	Cr1—N1—C8—C9	−2.6 (5)
N2^i^—Cr1—N1—C8	90.0 (3)	C5—C4—C8—N1	−0.8 (7)
N2—Cr1—N1—C8	1.9 (3)	C3—C4—C8—N1	−179.7 (4)
Cl1—Cr1—N1—C8	−86.9 (3)	C5—C4—C8—C9	179.2 (4)
Cl1^i^—Cr1—N1—C8	179.3 (3)	C3—C4—C8—C9	0.2 (7)
N1—Cr1—N2—C12	−177.2 (5)	C12—N2—C9—C1	−1.9 (7)
N1^i^—Cr1—N2—C12	10.7 (4)	Cr1—N2—C9—C1	−178.7 (4)
N2^i^—Cr1—N2—C12	90.7 (4)	C12—N2—C9—C8	176.5 (4)
Cl1—Cr1—N2—C12	−85.0 (4)	Cr1—N2—C9—C8	−0.3 (5)
Cl1^i^—Cr1—N2—C12	151.5 (11)	C10—C1—C9—N2	0.1 (7)
N1—Cr1—N2—C9	−0.8 (3)	C2—C1—C9—N2	177.4 (5)
N1^i^—Cr1—N2—C9	−172.9 (3)	C10—C1—C9—C8	−178.3 (5)
N2^i^—Cr1—N2—C9	−92.9 (3)	C2—C1—C9—C8	−1.0 (7)
Cl1—Cr1—N2—C9	91.4 (3)	N1—C8—C9—N2	2.0 (6)
Cl1^i^—Cr1—N2—C9	−32.1 (15)	C4—C8—C9—N2	−178.0 (4)
C10—C1—C2—C3	177.9 (5)	N1—C8—C9—C1	−179.6 (4)
C9—C1—C2—C3	0.8 (8)	C4—C8—C9—C1	0.5 (7)
C1—C2—C3—C4	−0.1 (9)	C9—C1—C10—C11	1.4 (8)
C2—C3—C4—C5	−179.3 (6)	C2—C1—C10—C11	−175.7 (6)
C2—C3—C4—C8	−0.4 (8)	C1—C10—C11—C12	−1.2 (9)
C8—C4—C5—C6	−0.2 (8)	C9—N2—C12—C11	2.2 (8)
C3—C4—C5—C6	178.7 (5)	Cr1—N2—C12—C11	178.4 (4)
C4—C5—C6—C7	1.4 (9)	C10—C11—C12—N2	−0.7 (9)
C8—N1—C7—C6	0.9 (7)		

Symmetry codes: (i) −*x*+1, *y*, −*z*+3/2.

## References

[bb1] Birk, T., Bendix, J. & Weihe, H. (2008). *Acta Cryst.* E**64**, m369–m370. 10.1107/S1600536808001153 PMC2960161 21201326

[bb2] Bruker (2000). *SMART* and *SAINT* Bruker AXS Inc., Madison, Wisconsin, USA.

[bb3] Sheldrick, G. M. (2000). *SADABS* University of Göttingen, Germany.

[bb4] Sheldrick, G. M. (2008). *Acta Cryst.* A**64**, 112–122. 10.1107/S0108767307043930 18156677

[bb5] Vincent, J. B. (2000). *Acc. Chem. Res.* **33**, 503–510. 10.1021/ar990073r 10913239

